# Point of care testing of Influenza A/B and RSV in an adult respiratory assessment unit is associated with improvement in isolation practices and reduction in hospital length of stay

**DOI:** 10.1099/jmm.0.001187

**Published:** 2020-04-06

**Authors:** Louise Berry, Louise Lansbury, Lydia Gale, Ann Marie Carroll, Wei Shen Lim

**Affiliations:** ^1^​ Department of Microbiology, Nottingham University Hospitals NHS Trust, Nottingham, UK; ^2^​ Division of Epidemiology and Public Health, University of Nottingham, Nottingham, UK; ^3^​ Department of Respiratory Medicine, Nottingham University Hospitals Trust, Nottingham, UK

**Keywords:** point-of-care test, respiratory tract infections, influenza, respiratory syncytial virus, length of stay, isolation

## Abstract

**Introduction.:**

Every winter seasonal influenza and other viral respiratory infections increase pressure on the health services and are associated with nosocomial infection and morbidity.

**Aim.:**

To compare provision of point-of-care (POC) testing with laboratory-based testing for influenza and RSV detection on an adult respiratory assessment unit to assess the impact on isolation practices and length of stay (LOS).

**Methodology.:**

Prospective interrupted ‘on-off’ study in adults admitted to the respiratory unit between December 2018 and April 2019 with a suspected respiratory tract infection. Nasopharyngeal samples were tested using either the GeneXpert rapid POC test for influenza and RSV (on-period), or were sent to the laboratory for multiplex PCR testing against a panel of 12 respiratory viruses (off-period). Outcome measures were time to patient isolation for infection control, LOS and turnaround time from admission to test results.

**Results.:**

Of 1145 patients evaluated, 755 were tested with POC and 390 with laboratory multiplex; a respiratory virus was identified in 164 (21.7 %) and 138 (35.4 %) patients respectively. A positive POC test was associated with a shorter time to isolation (mean difference 16.9 h, *P*<0.001), shorter LOS (mean difference 15.5 h, *P*=0.05,) and shorter turnaround time (mean difference 28.3 h, *P*<0.001), compared to laboratory testing.

**Conclusion.:**

Use of GeneXpert POC testing for Flu/RSV is associated with rapid reporting of results with significant improvements in isolation practices and reductions in LOS.

## Introduction

Influenza and other viral respiratory infections are common and put considerable pressure on the health services each winter in the UK, as well as burdening patients, families and carers, and having a wider socio-economic impact [1]. Some patients will be hospitalised as a result of their infection and a small number will die from complications [2].

There is a growing body of evidence that rapid detection of viral respiratory infections through testing patients as soon as possible after they are admitted to hospital allows more timely and targeted management of illness [3]. For example, antiviral drugs may be started more quickly and appropriately and unnecessary antibiotics stopped earlier [4–8]. Additionally, rapid diagnosis may offer the potential to decrease transmission to other patients and staff by rapid instigation of relevant infection prevention and control precautions [7, 9, 10], and may assist clinicians to make decisions earlier regarding discharge of infected patients, potentially reducing their length of stay in hospital and easing pressure on hospital beds [11].

The symptoms and signs of influenza may overlap with those seen with other respiratory pathogens, making diagnosis based on clinical presentation alone challenging [12]. Diagnostic testing is therefore useful to guide appropriate clinical management, with the current gold-standard being detection of viral RNA by reverse transcriptase polymerase chain reaction (RT-PCR), conducted in a specialised laboratory. However, although RT-PCR can produce a result within 4–8 h, transportation to the laboratory and batching of specimens the time to receipt of result may be considerable longer [13].

Point-of care (POC) tests are medical diagnostic tests performed by healthcare professionals on the ward who are trained in the use of the point-of-care analyser machines but are not laboratory staff. Results are available soon after testing. Second generation POC testing platforms using nucleic acid amplification technologies are now entering into clinical use and have been shown to have sensitivities and specificities of >90 % compared to first generation rapid influenza diagnostic tests [14].

One randomised controlled trial found an association with improved turnaround time, reduced length of stay and improved anti-viral use in the patient group which had point of care respiratory virus testing as opposed to standard lab testing [15]. Since then, in the UK, an increasing number of hospitals have started to introduce POC testing for respiratory viruses and some centres have reported their experiences [10, 16]. Most of these evaluations have been ‘before-after’ analyses focusing on length of stay, or hospital admission, as the primary outcome.

One of the expected benefits of POC testing is the more rapid availability of microbiological information to guide infection control decision-making and hence improve patient flow through hospital systems. Such benefits apply to both patients testing positive and those testing negative for respiratory viral infections. However, there are few published data on the magnitude of this benefit. We sought to determine the impact a PCR-based POC strategy for detecting respiratory viral infections on the institution of appropriate infection control measures and length of hospital stay, using an interrupted ‘on-off’ study design.

## Methods

We conducted a prospective interrupted ‘on-off’ study as part of a clinical evaluation programme comparing two strategies for detecting respiratory virus infections in adults presenting to hospital with symptoms of an acute respiratory tract infection (ARTI).

**Fig. 1.**
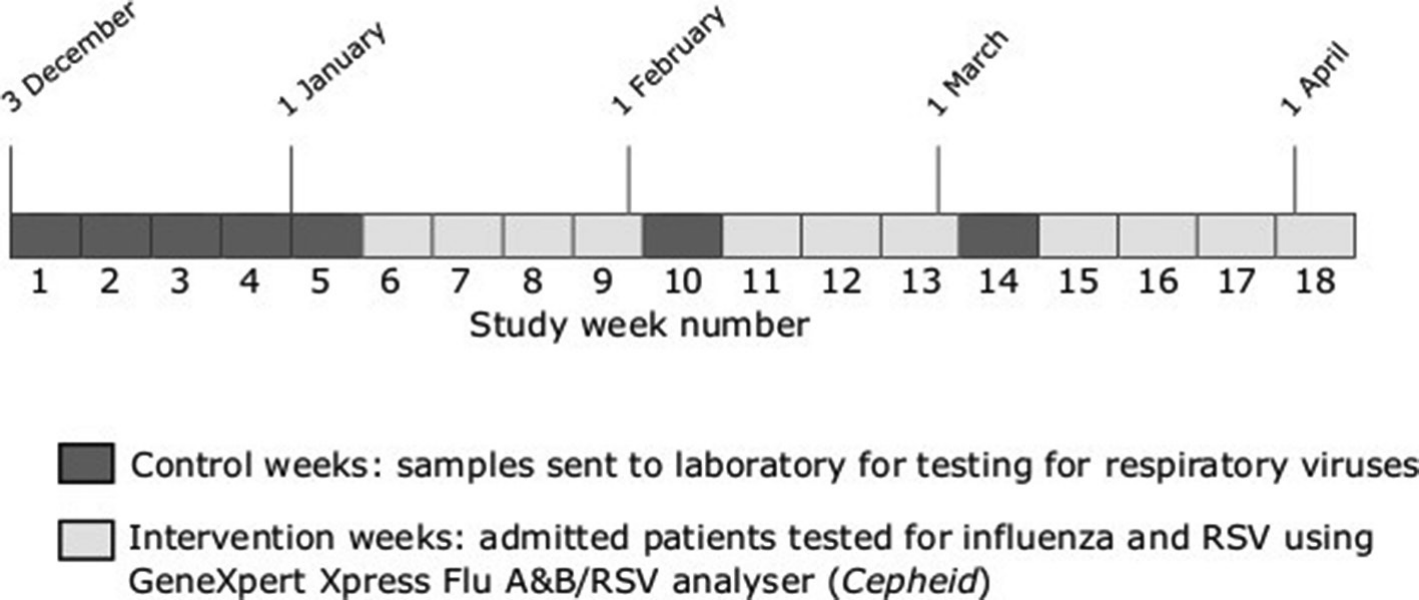
Timeline of the point-of-care and laboratory multiplex PCR respiratory virus testing periods.

**Fig. 2.**
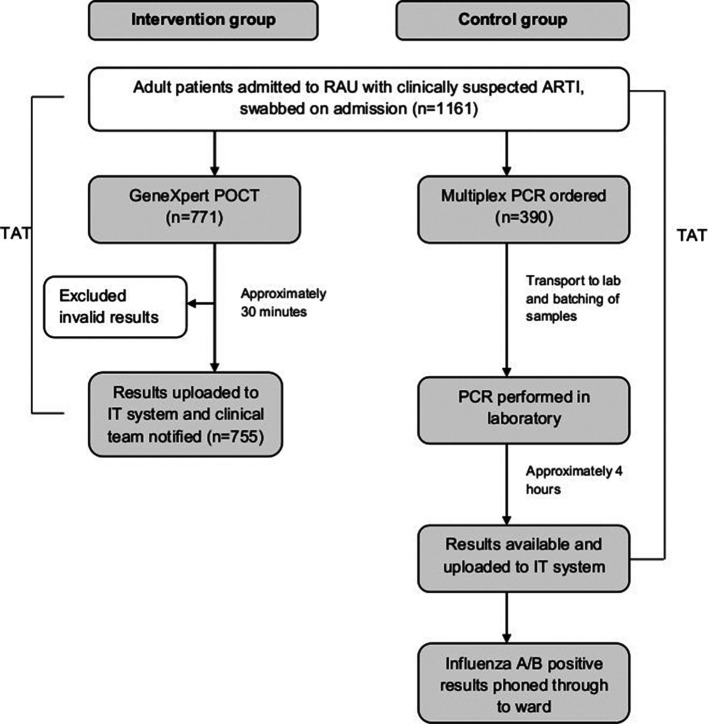
Flow chart of influenza testing during intervention and control periods RAU, Respiratory Assessment Unit; ARTI, Acute Respiratory Tract Infection; POCT, Point-of-care test; PCR, Polymerase Chain Reaction; TAT, Turnaround time.

Adults (aged >16 years) admitted to the Respiratory Assessment Unit (RAU) at a large teaching hospital between 3 December 2018 and 5 April 2019 with a clinically suspected ARTI had pharyngeal swabs taken as soon as possible after admission. The study period was divided into pre-specified control and intervention periods (7 and 11 weeks in total respectively)(Fig. 1). During the control period (weeks 1 to 5 (3 December 2018 to 6 Jan 2019), week 10 (4–10 February) and week 14 (4 to 10 March)), samples were sent to the microbiology laboratory for testing against a panel of 12 respiratory viruses using a commercially available multiplexed polymerase chain reaction (PCR) (AusDiagnostics, Sydney, Australia). During the study period there were approximately four to five PCR runs per day, depending upon the number of samples received. Diagnostic test results were automatically uploaded to hospital electronic microbiology records which were immediately accessible to clinical teams. In addition, positive influenza A/B results were telephoned through to clinical teams by microbiology staff, usually within 2 h of result availability. During intervention periods, samples were tested using a point-of-care (POC) strategy; an eight-cartridge GeneXpert Xpress Flu A and B/RSV analyser (Cepheid, Sunnyvale, CA, USA) was located on the RAU and samples were processed by trained ward staff according to manufacturer instructions. GeneXpert was available to use 24 h a day. The POC service was managed by the Pathology POC team and included initial verification of samples tested against the laboratory, ongoing weekly quality assurance testing, training of all Ward Authorised Trainers and other staff using a Standard Operating Procedures and Competency template, and continuing verification of results logged into the hospital and laboratory IT systems. Results were available within 30 min from introduction of the sample into the analyser and printed off as hard copies on RAU and placed in the front of medical notes for notification to the medical team (Fig. 2). For negative POC tests, multiplex PCR testing was only done when requested by a clinician and was not performed as a default. There was no test selection based upon the severity of symptoms or other patient factors during either the control or intervention periods.

For all patients, routinely recorded data were collected from hospital information systems regarding time from admission to result, length of stay and time to patient isolation in accordance with local infection control policies. This study was part of clinical evaluation of the implementation of a new diagnostic process within the hospital. Therefore, individual patient consent was not obtained; all data were anonymised prior to analysis.

From historical laboratory data collected during the previous two influenza seasons, it was predicted that 720 patients on the study ward would be tested by either POC test or in the laboratory during the study period. Based on an estimated mean length of stay of 123 h for laboratory-tested patients with a group standard deviation of 162 h and assuming a POCT to laboratory-testing ratio of 2.75 : 1, this sample size was estimated to have 80 % power to detect a 31 % decrease in mean length of stay in people tested by POCT with a significance level (alpha) of 0.05. However as this was not a clinical trial, we did not plan to recruit to a set sample size and all eligible patients who were tested during the study period were included in the analyses.

Length of stay was calculated in hours as the difference between the recorded time of admission and the time of discharge. For POCT, turnaround time was defined as the number of hours between admission to the ward and notification of the test result, and for laboratory-testing as the number of hours between admission to the ward and electronic transcription of the results. For patients who tested positive for a respiratory virus, the time to appropriate institution of infection control measures was calculated as the time from admission to the first recorded time of patient isolation either in a side-room, or in an ‘infection cohort bay’. Patients who were not isolated are henceforth described as being managed on an ‘open bay’.

### Statistical analysis

The frequency of test results were summarised for POC and laboratory-tested patients. Baseline Charlson co-morbidity index scores were compared between the groups using the two sample Wilcoxson rank-sum test. Continuous outcomes were analysed after transformation of the data to correct for right-skewed distribution. Length of stay and turnaround time data were log-transformed and time to instigation of control measures were cube-root transformed, with the decision on transformation type being made on the distribution of the data and numbers. The variance between the groups was compared using Levene’s test and the means of the laboratory-tested and POC-tested groups compared in an independent *t*-test with Satterthwaite’s correction for unequal variance if appropriate. Geometric means were obtained through back-transformation of data. For the length of stay analyses we used a linear regression model to adjust for Charlson co-morbidity index score. Subgroup analyses by test result were conducted.

For patients who were isolated immediately upon admission but who subsequently tested negative for a respiratory virus, the time to ‘de-isolation’ (move from isolation to an open bay) was estimated for the group as a whole. This calculation was based on the assumption that the proportions of positive and negative patients who were isolated immediately upon admission were similar and that patients were removed from isolation immediately upon receipt of a negative result.

All analyses were conducted in Stata 15.1.

## Results

In total, 1161 patients were admitted to RAU during the study period and tested for respiratory viruses. Excluding patients in whom test results were invalid (*n*=16), 1145 patients remained in the analyses; POC tested (*n*=755), laboratory tested (*n*=390). The median Charlson co-morbidity index scores were 4 (IQR 0–9) for POC tested patients and 4 (IQR 0–7) for those tested by the laboratory (*P*=0.15).

A respiratory virus was identified in 164 (21.7 %) of POC-tested patients and 138 (35.4 %) of laboratory-tested patients. Influenza virus was the predominant pathogen identified in both groups. The mean time from admission to availability of the result to the clinical team (turnaround time) was significantly shorter in the POC group compared to the control group (2.9 h (95 % CI 2.9 to 3.1) versus 31.2 h (95 % CI 29.6 to 32.9), *P*<0.001).

### Time to patient isolation

Overall, the mean time spent on the open bay prior to isolation was significantly shorter for patients who tested positive for influenza or RSV in the POC group compared to the laboratory-tested group (mean difference (MD) 16.9 hours *P*<0.001). On subgroup analysis according to pathogen, a significantly shorter mean time to isolation was noted for patients positive for influenza in the POC group compared to laboratory-tested group (MD 10.8 h, *P*<0.001). For RSV positive patients, there was no significant difference in time spent on the open ward between POC versus laboratory-tested groups (Table 1).

**Table 1.** Summary of Charlson co-morbidity index scores, turnaround times, and time to isolation for POC tested patients compared to laboratory tested patients

Result	Number (%) by test type		Median CCI (IQR)		Mean TAT (hours)*		*P* value	Mean time from open bay to isolation (hours)(95 % CI)		Mean difference (hours)*	*P* value
	POCT (*n*=755)	Lab (*n*=390)	POCT	Lab	POCT	Lab		POCT	Lab		
Influenza A/B	152 (20.1)	61 (15.6)	4 (0–4)	3 (0–4)	2.5 (2.2–2.9)	30.9 (27.0–35.3)	<0.001	3.05 (2.16–4.17)	13.81 (8.89–20.29)	10.76	<0.001
RSV	12 (1.6)	29 (7.4)	3.5 (0–10.5)	4 (0–13)	3.0 (1.4–6.5)	32.5 (35.6–41.2)	<0.001	35.06 (7.98–94.06)	43.01 (23.54–71.03)	7.95	0.73
Influenza A/B or RSV	164 (21.7)	90 (23.1)	4 (0–5)	3 (0–6)	2.5 (2.2–3.0)	31.4 (28.0–35.2)	<0.001	3.99 (2.76–5.54)	20.92 (14.53–28.94)	16.93	<0.001
Other	0 (0)†	48 (12.3)	n/a	3 (0–6)	n/a	39.1 (11.3–91.6)	n/a	n/a	n/a	n/a	n/a
Negative	591 (78.3)	252 (64.6)	4 (0–8)	4 (0–10)	3.0 (2.8–3.2)	29.8 (27.9–31.8)	<0.001	n/a	n/a	n/a	n/a

*Data were back-transformed.

†POC test only detects influenza A/B and RSV.

CCI, charlson morbidity index; CI, confidence intervals; IQR, interquartile range; Lab, multiplex PCR test; POCT, Point-of-care test; TAT, turnaround time.

In total, 35 of 252 (13.9 %) patients who eventually tested positive for a respiratory virus by either POC or in the lab, were isolated immediately upon admission. The proportion of influenza or RSV positive patients who were on the open ward 24 h or more after admission was significantly greater in the POC group (44.4 vs 8.6 %, odds ratio 8.4, 95 % CI 4.2 to 16.8, *P*<0.0001) (Table 2). Data on time to isolation for patients who eventually tested negative were not captured.

**Table 2.** Time to isolation for patients testing positive for influenza or RSV per test type

Time to isolation (hours)	POC tested: number isolated by the specified time point (%) *N*=162	Laboratory tested: number isolated by the specified time point (%) *N*=90	*P*-value (Chi-squared test)
0	30 (18.5)	5 (5.6)	0.004
≤1 h	44 (27.2)	16 (17.8)	0.09
≤6 h	102 (63.0)	24 (26.7)	<0.001
≤12 h	130 (80.2)	30 (33.3)	<0.001
≤24 h	148 (91.4)	50 (55.5)	<0.001
More than 24 h	14 (8.6)	40 (44.4)	<0.001

In a sensitivity analysis, assuming that the time to patient isolation is similar in those who eventually test negative as test positive, and that patients are ‘de-isolated‘ immediately upon receipt of a negative test result, POC testing potentially saved 2802 isolation hours (or 116.75 isolation bed-days) over the 18 week period, based on 140 laboratory-tested patients and 540 POC tested patients who were isolated within 24 h of admission but were eventually test negative. If patients were isolated immediately upon admission, there was a potential saving of 3198 isolation hours (or 133 isolation bed-days) over the 18 week period based on 117 patients who were isolated immediately upon admission but were eventually test negative.

### Length of stay

The mean length of stay for all patients admitted and tested for respiratory viruses during the study period was 129.1 h (SD 167.6), median 77 h (IQR 32–157). Patients who had a respiratory virus detected by either test had an average LOS that was significantly shorter than those in whom no respiratory virus was found (adjusted means for negative and positive patients 74.7 h (95 % CI 69.1 to 80.9) versus 56.7 (95 % CI 49.7 to 64.7), *P*<0.01, respectively), Table 3.

**Table 3.** Length of stay in POC and laboratory-tested groups according to test results

Comparison	Number included in analysis	Unadjusted MD LOS (hours)	*P* value	Adjusted MD LOS*	*P* value
**Positive vs negative result**					
Any test (Laboratory/POCT)	1144	−20.1	<0.01	−18.1	<0.01
POCT only	755	−26.5	<0.01	−24.9	<0.01
Laboratory only	389	−11.2	0.17	−8.7	0.28
**Influenza positive vs negative**					
Any test (Laboratory/POCT)	1055			−22.36	<0.01
POCT only	743			−26.68	<0.01
Laboratory tested only	312			−9.7	0.37
**RSV positive vs negative**					
Any test (Laboratory/POCT)	883			−.4.6	0.73
POCT only	603			4.7	0.86
Laboratory tested only	280			−8.6	0.73
**POCT vs laboratory**					
Any result (positive or negative)	1144	−1.622	0.75	−3.38	0.51
Any positive	302	−14.4	0.0639	−15.5	0.05
Influenza positive only	213			−16.3	0.11
RSV positive only				12.7	0.68
Negative	842	−1.0	0.88	0.97	0.94

*Adjusted for Charlson morbidity index

MD, mean difference; LOS, length of stay.

Patients who tested positive in the POC group had a shorter LOS compared to those in the laboratory-tested group (adjusted mean length of stay 48.8 h (95 % CI 52.6 to 64.3) v 64.3 h (95 % CI 40.6 to 58.5), *P*=0.05).

Within the POC group, those who tested positive for either influenza or RSV were discharged on average 25 h earlier than those who tested negative (adjusted means 50.3 (95 % CI 41.8 to 60.5) versus 75.2 (95 % CI 68.3 to 82.9), *P*<0.01, respectively). Patients positive for influenza were discharged on average almost 27 h earlier than influenza-negative patients (adjusted mean 48.5 (95 % CI 40.1 to 26.7) versus 75.2 (95 % CI 68.3 to 82.9), *P*<0.01, respectively).

Within the laboratory-tested group, there was no significant difference in LOS between patients who tested positive for respiratory viruses and those who tested negative.

Based on the observation that patients with a positive result in the POC group were discharged approximately 15.5 h earlier than corresponding patients in the laboratory-tested group (*P*=0.05), an estimated 106 bed-days were saved in total during the POC ‘on-period’ of 18 weeks (equivalent to 9.6 bed-days per week). A comparable difference in mean LOS was also seen in a sensitivity analysis in which only patients who were admitted during the peak influenza season (weeks 51 to 13) were included (*n*=261, adjusted mean difference 18.6 h, *P*=0.03).

## Discussion

The main finding from our study is that implementation of POC testing delivered benefits to patient care admitted to hospital with respiratory illness in terms of more rapid confirmation of infection with influenza and RSV, a shorter length of stay and more rapid isolation of patients testing positive for influenza or RSV.

### Time to isolation

The reduction in time to isolation, either in a single use side room or a cohort bay for influenza positive patients, during periods when POC was operational, demonstrates the benefit of rapid diagnostics for infection prevention and control of respiratory viruses. The time to isolation of POC influenza positive patients was close to that of the turnaround time to results (~3 h). The mean time to isolation of laboratory tested influenza positive patients was 13 h whereas the mean turnaround time of result was 31 h. Thus, many patients are isolated empirically whilst awaiting results. POC allowed earlier release of isolation beds for negative patients allowing more efficient use of limited isolation resources. Our study indicates that the introduction of POC testing could potentially save between 6.5 to 7.5 isolation bed-days per week during the winter. The mean time to isolation for of RSV positive patients was not statistically different and was 35 h in the POC group. This likely reflected a prioritisation of isolation facilities for influenza positive patients within the setting on an adult respiratory ward, given the relative severity of its impact on morbidity and clinical sequelae.

Isolation facilities such as side rooms are at a premium in winter months when a large number of patients with respiratory symptoms are admitted to hospital. Frequently there are insufficient side rooms available to isolate all patients suspected to have a respiratory virus infection and there are also requirements to isolate those with other infections such as carbapenem-resistant Enterobacteriaceae, methicillin-resistant *Staphylococcus aureus* and *Clostridium difficile* infection.

Rapid point of care testing allows the rapid identification of those with confirmed infection and for the more judicious use of side rooms, personal protective equipment and anti-virals. Previous studies [15, 17, 18] have demonstrated the utility of POCT to de-isolate patients more promptly when not required. Rapid isolation is important for effective infection control within the hospital. In previous years we have seen the consequences of poor isolation practice with increased use of oseltamivir for post-exposure prophylaxis and nosocomial transmissions within the trust. In 2017/8 this was estimated to be 15 % of influenza cases diagnosed during the influenza season. A recent study using phylogenetic analysis of influenza isolates demonstrated genetic clustering in 15.8 % of hospital influenza cases indicating nosocomial acquisition [19]. Nosocomial acquisition of influenza has knock-on effects in terms of morbidity and delays to discharge, the financial impact of which has not been clarified and further studies are needed to understand the economic effects of these aspects in more detail. A previous study of the introduction of POC testing in the emergency department together with a dedicated influenza ward demonstrated benefits in terms of reduction of hospital acquired influenza [10].

Implementation of the rapid POC test was well received by staff and patients alike who, when surveyed all reported wanting the service to be available in future years. Patient feedback received was universally positive, with many citing a reduction in anxiety due to the uncertainty of their diagnosis and improved confidence in the care they receive. Clinicians noted the benefits of POC testing in terms of aiding clinical decision making around anti-viral and antimicrobial use, although this was not formally assessed in our study. Other studies have formally assessed the benefits of POC testing in terms of more timely and judicious use of anti-viral prescriptions [20] antibiotic usage [15, 21, 22] and other microbiological investigations [23].

### Length of stay

Several studies have shown that POCT for viral respiratory infections is associated with multiple benefits, including a shorter length of stay for those testing positive [15, 21, 23, 24]. Possible explanations include improved clinician confidence in discharging patients with a confirmed respiratory virus infection and the association between earlier anti-viral initiation and shortened hospitalisation [25].

The reduction in LOS has important economic benefits. In terms of bed-day savings this equated to ~nine bed-days per week which over an 18 week flu season could translate to approximately £48 600 (based on an estimate of £300 per bed-day). In addition patient flow is optimised as bed-days saved can help with pressure in the emergency department (ED) which faces time constraints to transfer patients onto admitting wards, in order to free up bed capacity. A previous study utilizing the GeneXpert platform for influenza estimated reductions in costs of testing and treating patients by 103€ per ED patient and 64€ per hospitalized patient [26].

### Turnaround time

The reduction in turnaround time with POC testing has been well documented in previous studies [15, 23, 24] and in our study this difference likely reflects the time spent for the specimen to reach the centralised laboratory on a different hospital site and the fact that samples are batch tested in the laboratory at regular intervals within laboratory working hours. We took the turnaround time to be the time between sampling and availability of the result to the clinical team, not the time at which results were phoned through to the ward, so it is unlikely that the speed of this process could be increased.

### Strengths and limitations of study

The strengths of this study include its utilization of real-world routinely collected clinical data which enhances the generalisability and reproducibility of our findings. We specifically restricted our analyses to one patient group – those admitted with primary respiratory complaints rather than include those from other patient groups such as haematology and oncology patients whose teams were also able to access the point of care testing service. In addition, we conducted the study over one influenza season to reduce the effect of cofounding variables such as type of circulating influenza and prevalence of influenza between subsequent years.

Due to lack of randomisation there is potential for bias in terms of the time in which POC was utilised in the course of the winter season. The control period was mainly when RSV was the predominant virus and intervention period mainly when influenza was circulating, which may have introduced bias in terms of outcomes. Sensitivity analysis restricted to admissions during the weeks when influenza hospitalisations were above baseline threshold levels confirmed the significantly decreased mean LOS in POC tested patients. Although results of outcomes studied are similar when adjusted for type of pathogen, isolation facilities may have been under different levels of constraint at different time points in the flu season. It is notable that a larger percentage of patients in the POC group were isolated immediately upon admission and there may have been a bias to those who were quickly suspected to have and tested for influenza.

We utilised an interrupted series design that was powered for differences in length of stay primarily. This meant that the control vs intervention periods were of unequal lengths and at differing time points in the influenza season. In addition, we did not assess the impact that POC testing had on time to initiation of anti-virals or reduction in antibiotic use, as these outcomes have been reported previously by other investigators.

Although our study suggests a benefit in terms of more rapid isolation of patients with influenza, a further study would be required to assess the impact in terms of a reduction of onward transmission in this particular setting.

## Conclusion

Introduction of a POC testing strategy for influenza and RSV infection in adults admitted with acute respiratory illness was associated with more rapid reporting of results, more appropriate isolation practices and reductions in LOS compared to laboratory testing.
